# Crystal structure of (*E*)-pent-2-enoic acid

**DOI:** 10.1107/S2056989015007203

**Published:** 2015-04-18

**Authors:** Tim Peppel, Marcel Sonneck, Anke Spannenberg, Sebastian Wohlrab

**Affiliations:** a Leibniz-Institut für Katalyse e. V. an der Universität Rostock, Albert-Einstein-Strasse 29a, 18059 Rostock, Germany

**Keywords:** crystal structure, hydrogen bond, dimer, unsaturated carb­oxy­lic acid

## Abstract

The mol­ecule of the title compound, C_5_H_8_O_2_, a low-melting α,β-unsaturated carb­oxy­lic acid, is essentially planar [maximum displacement = 0.0239 (13) Å]. In the crystal, mol­ecules are linked into centrosymmetric dimers *via* pairs of O—H⋯O hydrogen bonds.

## Related literature   

For the synthesis of unsaturated carb­oxy­lic acids including the title compound, see: Shabtai *et al.* (1981 ▸); Gastaminza *et al.* (1984 ▸); Outurquin & Paulmier (1989 ▸). For crystal structure determinations of acrylic acid, see: Higgs & Sass (1963 ▸); Chatani *et al.* (1963 ▸); Boese *et al.* (1999 ▸); Oswald & Urquhart (2011 ▸). For the structure of crotonic acid, see: Shimizu *et al.* (1974 ▸). For the structure of related hexenoic acid cocrystals, see: Aakeröy *et al.* (2003 ▸); Stanton & Bak (2008 ▸).
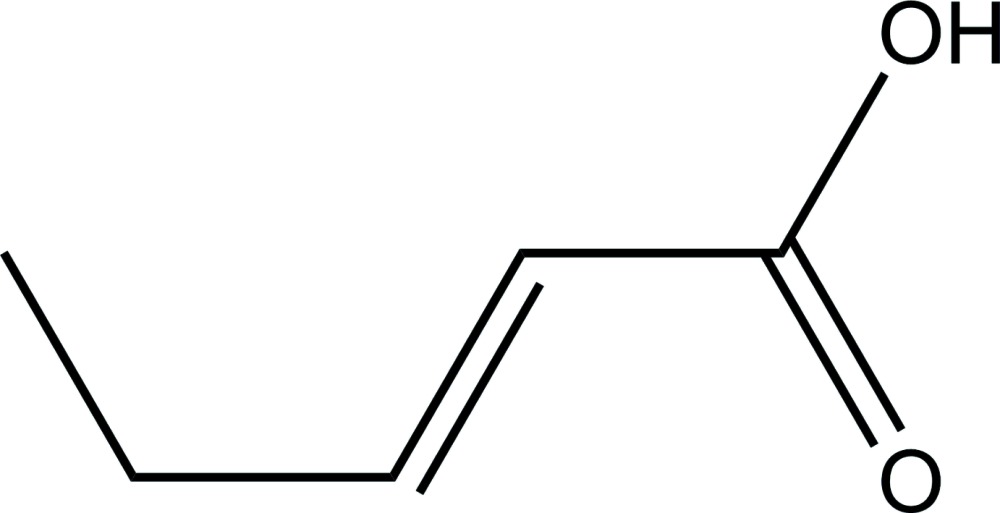


## Experimental   

### Crystal data  

C_5_H_8_O_2_
*M* *_r_* = 100.11
Triclinic, 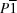

*a* = 6.7336 (13) Å
*b* = 6.7821 (13) Å
*c* = 7.2349 (14) Å
α = 67.743 (2)°
β = 75.518 (2)°
γ = 64.401 (2)°
*V* = 274.29 (9) Å^3^
*Z* = 2
Mo *K*α radiation
μ = 0.09 mm^−1^
*T* = 150 K
0.51 × 0.35 × 0.27 mm

### Data collection  

Bruker APEXII CCD diffractometer
Absorption correction: multi-scan (*SADABS*; Bruker, 2014 ▸) *T*_min_ = 0.81, *T*_max_ = 0.97
7544 measured reflections
1323 independent reflections
1122 reflections with *I* > 2σ(*I*)
*R*_int_ = 0.026

### Refinement  

*R*[*F* ^2^ > 2σ(*F* ^2^)] = 0.037
*wR*(*F* ^2^) = 0.113
*S* = 1.10
1323 reflections
69 parameters
H atoms treated by a mixture of independent and constrained refinement
Δρ_max_ = 0.36 e Å^−3^
Δρ_min_ = −0.19 e Å^−3^

### 

Data collection: *APEX2* (Bruker, 2014 ▸); cell refinement: *SAINT* (Bruker, 2013 ▸); data reduction: *SAINT*; program(s) used to solve structure: *SHELXS97* (Sheldrick, 2008 ▸); program(s) used to refine structure: *SHELXL2014* (Sheldrick, 2015 ▸); molecular graphics: *SHELXL2014*; software used to prepare material for publication: *SHELXL2014*.

## Supplementary Material

Crystal structure: contains datablock(s) I, New_Global_Publ_Block. DOI: 10.1107/S2056989015007203/rz5155sup1.cif

Structure factors: contains datablock(s) I. DOI: 10.1107/S2056989015007203/rz5155Isup2.hkl

Supporting information file. DOI: 10.1107/S2056989015007203/rz5155Isup3.cml

**Fig. 1.**
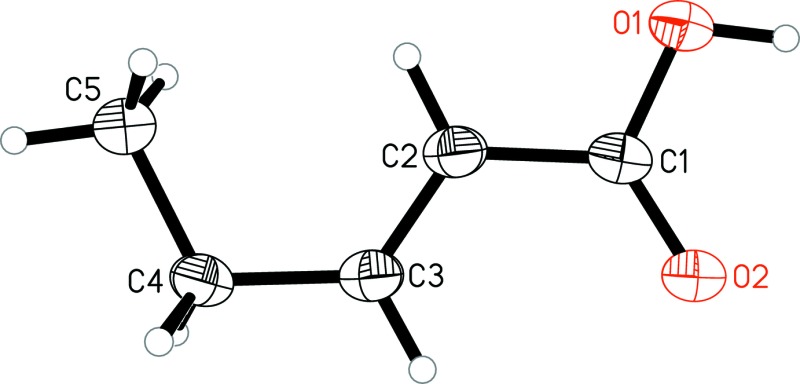
The molecular structure of the title compound with displacement ellipsoids drawn at 50% probability level.

. DOI: 10.1107/S2056989015007203/rz5155fig1.tif
The mol­ecular structure of the title compound with displacement ellipsoids drawn at 50% probability level.

**Fig. 2.**
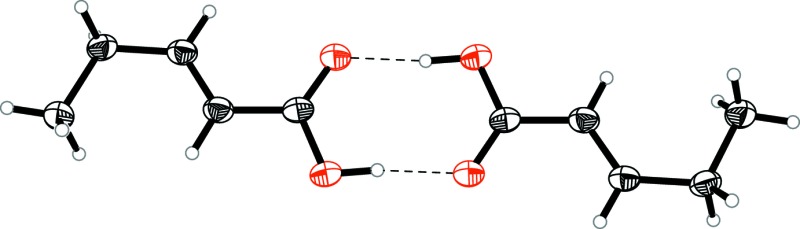
ORTEP representation of a dimer formed by intermolecular O—H···O hydrogen bonds.

ORTEP . DOI: 10.1107/S2056989015007203/rz5155fig2.tif
*ORTEP* representation of a dimer formed by inter­molecular O—H⋯O hydrogen bonds.

CCDC reference: 1058870

Additional supporting information:  crystallographic information; 3D view; checkCIF report

## Figures and Tables

**Table 1:** Hydrogen-bond geometry (, )

*D*H*A*	*D*H	H*A*	*D* *A*	*D*H*A*
O1H1O2^i^	0.95(2)	1.69(2)	2.6322(13)	173.3(19)

Symmetry code: (i) 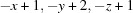.

## supplementary crystallographic information

### S1. Synthesis and crystallization

Malonic acid (24.8 g, 237.8 mmol, 1eq) was dissolved in dry pyridine (37.6 g, 475.7 mmol, 2 eq) at room temperature in a three-necked flask equipped with a magnetic stir bar and a reflux condenser under a mild flow of argon. Propanal (13.8 g, 240.2 mmol, 1 eq) was then added in one portion and the resulting clear solution further stirred for 72 h at room temperature under argon. Afterwards, the resulting light yellow to orange solution was brought to an acidic pH value by adding phospho­ric acid at 0°C (42.5 wt.-%, 582.7 mmol, 2.45 eq). The resulting two layers were extracted three times with 150 mL portions of ethyl acetate and reduced to a volume of ca. 150 mL. To remove impurities from aldol condensation the raw acid was converted into the corresponding sodium salt by addition of an aqueous solution of sodium carbonate (18.9 g, 178.4 mmol, 0.75 eq in 200 mL). After stirring for 30 minutes the water phase was separated und extracted three times with 150 mL portions of ethyl acetate. The water phase was then acidified with concentrated hydro­chloric acid (35.2 g, 356.7 mmol, 1.5 eq), the organic phase was separated and the water phase was again extracted three times with 150 mL portions of ethyl acetate. The combined organic phases were dried over Na_2_SO_4_ and evaporated to dryness under diminished pressure. The resulting raw product was further purified by distillation *in vacuo* yielding the product in purity >99% (GC). M. p. 10°C. ^1^H NMR (400 MHz, CDCl_3_): *δ* = 12.35 (br s, 1H, OH); 7.14 (dt, ^3^*J* = 15.6 Hz, ^3^*J* = 6.3 Hz, 1H, -CH-); 5.82 (dt, ^3^*J* = 15.6 Hz, ^4^*J* = 1.7 Hz, 1H, -CH-); 2.30-2.21 (m, 2H, -CH_2_-); 1.08 (t, ^3^*J* = 7.4 Hz, 3H, -CH_3_-). ^13^C NMR (100 MHz, CDCl_3_): *δ* = 172.69 (CO); 153.77 (CH); 119.76 (CH); 25.54 (CH_2_); 21.10 (CH_3_). MS (EI, 70 eV): *m/z* = 100 (M^+^, 50), 83 (13), 82 (23), 81 (10), 58 (11), 57 (17), 56 (23), 55 (100), 54 (43), 53 (35), 52 (12), 51 (25), 50 (28), 45 (77), 41 (36), 40 (13), 39 (99), 38 (25), 37 (11), 29 (61). HRMS (ESI-TOF/MS): calculated for C_5_H_8_O_2_ (M^+^) 99.04515, found 99.04529. Elemental analysis for C_5_H_8_O_2_ % (calc.): C 59.99 (59.98); H 8.05 (8.05). Suitable single crystals were grown by slow evaporation of an ethano­lic solution at -30 °C over one week.

### S2. Refinement

The carb­oxy­lic H atom could be found in a difference Fourier map and was refined freely. All other H atoms were placed in idealized positions with d(C—H) = 0.95 Å (CH), 0.99 Å (CH_2_), 0.98 Å (CH_3_) and refined using a riding model with *U*_iso_(H) fixed at 1.2 *U*_eq_(C) for CH and CH_2_ and 1.5 *U*_eq_(C) for CH_3_. A rotating model was used for the methyl group.

### Crystal data

**Table:**

C_5_H_8_O_2_	*Z* = 2
*M**_r_* = 100.11	*F*(000) = 108
Triclinic, *P*1	*D*_x_ = 1.212 Mg m^−^^3^
*a* = 6.7336 (13) Å	Mo *K*α radiation, λ = 0.71073 Å
*b* = 6.7821 (13) Å	Cell parameters from 4399 reflections
*c* = 7.2349 (14) Å	θ = 3.1–28.7°
α = 67.743 (2)°	µ = 0.09 mm^−^^1^
β = 75.518 (2)°	*T* = 150 K
γ = 64.401 (2)°	Prism, colourless
*V* = 274.29 (9) Å^3^	0.51 × 0.35 × 0.27 mm

### Data collection

**Table:**

Bruker APEXII CCD diffractometer	1323 independent reflections
Radiation source: fine-focus sealed tube	1122 reflections with *I* > 2σ(*I*)
Detector resolution: 8.3333 pixels mm^-1^	*R*_int_ = 0.026
φ and ω scans	θ_max_ = 28.0°, θ_min_ = 3.1°
Absorption correction: multi-scan (*SADABS*; Bruker, 2014)	*h* = −8→8
*T*_min_ = 0.81, *T*_max_ = 0.97	*k* = −8→8
7544 measured reflections	*l* = −9→9

### Refinement

**Table:**

Refinement on *F*^2^	0 restraints
Least-squares matrix: full	Hydrogen site location: mixed
*R*[*F*^2^ > 2σ(*F*^2^)] = 0.037	H atoms treated by a mixture of independent and constrained refinement
*wR*(*F*^2^) = 0.113	*w* = 1/[σ^2^(*F*_o_^2^) + (0.0562*P*)^2^ + 0.0602*P*] where *P* = (*F*_o_^2^ + 2*F*_c_^2^)/3
*S* = 1.10	(Δ/σ)_max_ < 0.001
1323 reflections	Δρ_max_ = 0.36 e Å^−^^3^
69 parameters	Δρ_min_ = −0.19 e Å^−^^3^

### Special details

**Table:**

Geometry. All e.s.d.'s (except the e.s.d. in the dihedral angle between two l.s. planes) are estimated using the full covariance matrix. The cell e.s.d.'s are taken into account individually in the estimation of e.s.d.'s in distances, angles and torsion angles; correlations between e.s.d.'s in cell parameters are only used when they are defined by crystal symmetry. An approximate (isotropic) treatment of cell e.s.d.'s is used for estimating e.s.d.'s involving l.s. planes.

### Fractional atomic coordinates and isotropic or equivalent isotropic displacement parameters (Å^2^)

**Table:**

	*x*	*y*	*z*	*U*_iso_*/*U*_eq_	
C1	0.36613 (18)	0.77456 (19)	0.59878 (16)	0.0284 (3)	
C2	0.25933 (18)	0.6065 (2)	0.66526 (16)	0.0295 (3)	
H2	0.1507	0.6289	0.5893	0.035*	
C3	0.31028 (18)	0.4243 (2)	0.82842 (16)	0.0290 (3)	
H3	0.4190	0.4066	0.9018	0.035*	
C4	0.2111 (2)	0.2446 (2)	0.90640 (17)	0.0323 (3)	
H4A	0.1414	0.2401	1.0450	0.039*	
H4B	0.3320	0.0930	0.9135	0.039*	
C5	0.0389 (2)	0.2791 (2)	0.78294 (19)	0.0372 (3)	
H5A	−0.0864	0.4244	0.7812	0.056*	
H5B	−0.0133	0.1517	0.8430	0.056*	
H5C	0.1055	0.2834	0.6452	0.056*	
O1	0.30172 (15)	0.93766 (15)	0.42861 (12)	0.0357 (3)	
O2	0.50223 (14)	0.76619 (15)	0.69113 (12)	0.0361 (3)	
H1	0.376 (3)	1.039 (4)	0.395 (3)	0.071 (6)*	

### Atomic displacement parameters (Å^2^)

**Table:**

	*U*^11^	*U*^22^	*U*^33^	*U*^12^	*U*^13^	*U*^23^
C1	0.0277 (5)	0.0275 (6)	0.0251 (5)	−0.0060 (4)	−0.0070 (4)	−0.0056 (4)
C2	0.0270 (5)	0.0326 (6)	0.0272 (5)	−0.0093 (5)	−0.0077 (4)	−0.0066 (4)
C3	0.0265 (5)	0.0325 (6)	0.0257 (5)	−0.0086 (4)	−0.0067 (4)	−0.0071 (4)
C4	0.0321 (6)	0.0332 (6)	0.0267 (5)	−0.0121 (5)	−0.0087 (4)	−0.0010 (4)
C5	0.0378 (6)	0.0382 (7)	0.0355 (6)	−0.0177 (5)	−0.0135 (5)	−0.0013 (5)
O1	0.0406 (5)	0.0339 (5)	0.0295 (4)	−0.0147 (4)	−0.0153 (3)	0.0015 (3)
O2	0.0402 (5)	0.0345 (5)	0.0332 (5)	−0.0155 (4)	−0.0166 (4)	−0.0005 (3)

### Geometric parameters (Å, º)

**Table:**

C1—O2	1.2337 (14)	C4—C5	1.5239 (16)
C1—O1	1.3223 (13)	C4—H4A	0.9900
C1—C2	1.4723 (16)	C4—H4B	0.9900
C2—C3	1.3301 (16)	C5—H5A	0.9800
C2—H2	0.9500	C5—H5B	0.9800
C3—C4	1.4981 (16)	C5—H5C	0.9800
C3—H3	0.9500	O1—H1	0.95 (2)
			
O2—C1—O1	122.75 (11)	C5—C4—H4A	108.4
O2—C1—C2	123.99 (10)	C3—C4—H4B	108.4
O1—C1—C2	113.26 (10)	C5—C4—H4B	108.4
C3—C2—C1	122.03 (10)	H4A—C4—H4B	107.5
C3—C2—H2	119.0	C4—C5—H5A	109.5
C1—C2—H2	119.0	C4—C5—H5B	109.5
C2—C3—C4	125.63 (10)	H5A—C5—H5B	109.5
C2—C3—H3	117.2	C4—C5—H5C	109.5
C4—C3—H3	117.2	H5A—C5—H5C	109.5
C3—C4—C5	115.33 (10)	H5B—C5—H5C	109.5
C3—C4—H4A	108.4	C1—O1—H1	108.7 (12)
			
O2—C1—C2—C3	−3.05 (19)	C1—C2—C3—C4	−179.72 (10)
O1—C1—C2—C3	176.98 (11)	C2—C3—C4—C5	0.45 (18)

### Hydrogen-bond geometry (Å, º)

**Table:**

*D*—H···*A*	*D*—H	H···*A*	*D*···*A*	*D*—H···*A*
O1—H1···O2^i^	0.95 (2)	1.69 (2)	2.6322 (13)	173.3 (19)

Symmetry code: (i) −*x*+1, −*y*+2, −*z*+1.
