# Supplementary figures and images for: Crystal structure of (E)-pent-2-enoic acid
Source: Acta Crystallogr E Crystallogr Commun. 2015 Apr 18;71(Pt 5):o316. doi: 10.1107/S2056989015007203 (PMC4420089; doi:10.1107/S2056989015007203)

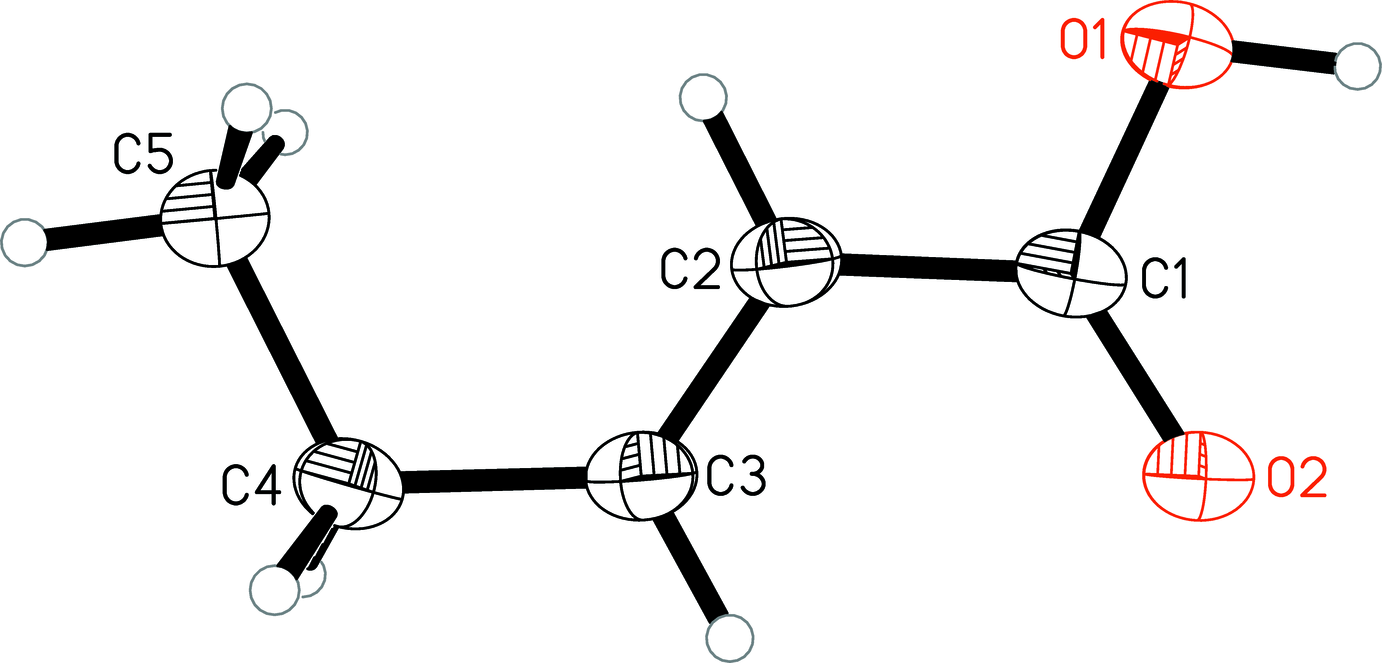

Supplement: Supplementary file 4 [file e-71-0o316-fig1.tif]

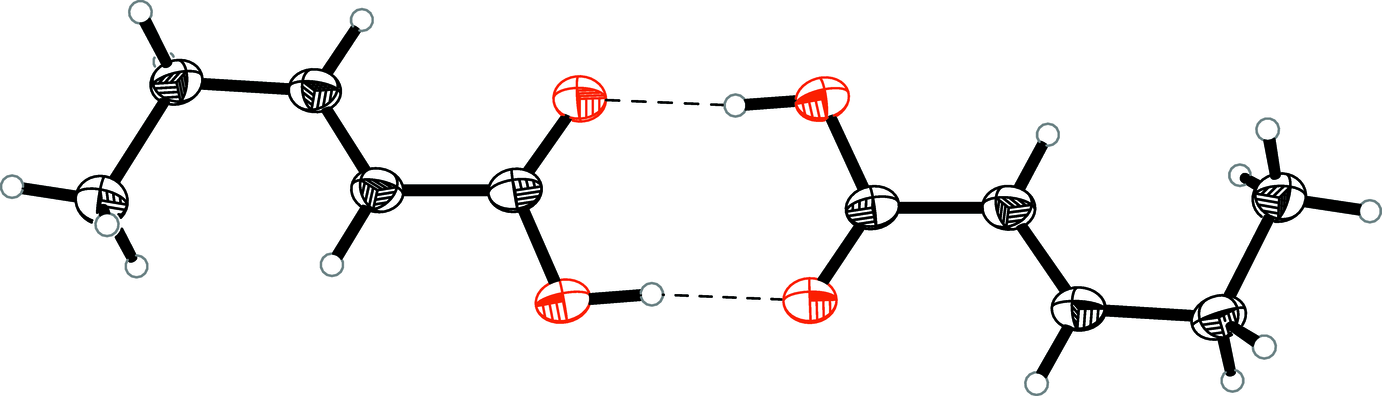

Supplement: Supplementary file 5 [file e-71-0o316-fig2.tif]
